# Managing metabolic syndrome in bipolar disorder: multidisciplinary monitoring and treatment strategies

**DOI:** 10.3389/fpsyt.2026.1699908

**Published:** 2026-04-22

**Authors:** Jorge C. Correia, Helene Richard-Lepouriel, Anne Chalut-Carpentier, Zoltan Pataky

**Affiliations:** 1 Unit of Therapeutic Patient Education, WHO Collaborating Centre, Service of Primary Care Medicine, Geneva University Hospitals, Geneva, Switzerland; 2 Faculty Diabetes Centre, Faculty of Medicine, University of Geneva, Geneva, Switzerland; 3 Mood Disorder and Anxiety Unit, Psychiatric Specialties Service, Department of Psychiatry, Geneva University Hospitals, Geneva, Switzerland; 4 Department of Psychiatry, University of Geneva, Geneva, Switzerland; 5 Centre Métabolique Nutrition et Mouvement, Geneva, Switzerland

**Keywords:** bipolar disorder, inflammation, integrated care, lifestyle intervention, metabolic syndrome, psychotropic medications, therapeutic education

## Abstract

**Background:**

Bipolar disorder (BD) is a chronic psychiatric illness associated with high rates of medical comorbidities, among which metabolic syndrome (MetS) is particularly prevalent and consequential. Affecting nearly half of individuals with BD, MetS compounds the risk of cardiovascular disease, type 2 diabetes, and premature mortality, while also undermining psychiatric stability and cognitive functioning. Despite these outcomes, metabolic health remains underrecognized and undertreated in psychiatric care.

**Objective:**

This narrative review aims to examine the bidirectional relationship between BD and metabolic syndrome and to highlight multidisciplinary strategies for metabolic monitoring and clinical management in this population.

**Methods:**

A targeted literature search was conducted using PubMed (2000–2025), covering studies on the epidemiology, behavioral and biological mechanisms, pharmacologic and lifestyle interventions, and clinical care integration related to BD and MetS. The review followed established quality guidance for narrative synthesis and was structured using the Population–Concept–Context framework to improve transparency in the selection and synthesis of the literature.

**Results:**

The comorbidity between BD and MetS is shaped by multiple interacting factors, including shared behavioral risk factors, inflammatory pathways, hypothalamic–pituitary–adrenal (HPA) axis dysregulation, and the metabolic effects of psychotropic medications. While pharmacological treatment remains essential for mood stabilization, systematic metabolic monitoring is crucial to mitigate treatment-related risks. Evidence supports the central role of lifestyle interventions, including nutritional strategies and physical activity in reducing cardiometabolic risk. Emerging therapeutic approaches such as GLP-1 receptor agonists and ketogenic metabolic therapy show potential benefits but require careful clinical integration. In selected individuals with severe or refractory obesity, bariatric surgery may be considered. Therapeutic patient education (TPE) represents the cornerstone of care by supporting self-management, treatment adherence, and shared decision-making.

**Conclusion:**

Addressing the dual burden of BD and MetS requires a multidisciplinary and patient-centered approach integrating metabolic monitoring, lifestyle interventions, pharmacological strategies, and therapeutic patient education. Strengthening collaboration between psychiatry, primary care, and metabolic specialists is essential to reduce cardiometabolic risk and improve long-term health outcomes in this vulnerable population.

## 1 Introduction

Bipolar disorder (BD) is a chronic, relapsing psychiatric illness characterized by recurrent episodes of mania or hypomania and depression, interspersed with periods of euthymia. It affects approximately 1–2% of the global population, with onset typically in late adolescence or early adulthood (1, 2). The disorder is associated with substantial functional impairment, elevated suicide risk, and increased medical comorbidity, contributing to a 10–20 year reduction in life expectancy on average (3–5).

Among the most clinically significant medical comorbidities is metabolic syndrome (MetS), a cluster of interrelated cardiometabolic abnormalities that includes abdominal obesity, hyperglycemia, elevated blood pressure, hypertriglyceridemia, and reduced HDL cholesterol (6, 7). When present, MetS confers a markedly increased risk of developing type 2 diabetes mellitus, cardiovascular disease, and premature death (8). It affects roughly 25% of the general population, but prevalence estimates rise to 40–50% among individuals with BD, highlighting a substantial cardiometabolic burden in this population (9–11).

This high comorbidity has profound consequences. The presence of MetS in people living with bipolar disorder (PlwBD) has been associated with greater cognitive impairment, lower treatment response, poorer quality of life, and increased rates of hospitalization and mortality (12–14). Multiple factors likely contribute to this association, including behavioral factors such as irregular eating patterns, low physical activity, substance use, and sleep disruption (15). Compounding these risks are the iatrogenic effects of psychotropic medications, particularly second-generation antipsychotics and mood stabilizers, which can induce weight gain and metabolic dysregulation (16–18).

Despite this, metabolic health is often under-monitored and undertreated in psychiatric settings (19). This gap in care reflects fragmented service delivery and limited integration between psychiatric and somatic health care, despite increasing awareness of the complex interactions between mental and physical health (20).

The goal of this narrative review is to synthesize current evidence on the pathophysiological mechanisms that link BD and MetS and to discuss practical strategies for metabolic monitoring and multidisciplinary management. By drawing on research across psychiatry, endocrinology, nutrition, and behavioral science, this review aims to support a more integrated model of care for this vulnerable population.

## 2 Methodological approach

This article was conducted as a narrative review aimed at synthesizing current evidence on the relationship between bipolar disorder (BD) and metabolic syndrome (MetS), with a particular focus on mechanisms underlying their co-occurrence and on clinical strategies for monitoring and management.

To structure the review, we followed the Population–Concept–Context (PCC) framework recommended by the Joanna Briggs Institute (JBI) for narrative and scoping reviews (21).

- Population: people living with bipolar disorder (PlwBD).
- Concept: metabolic syndrome and related cardiometabolic disturbances, including obesity, insulin resistance, and dyslipidemia.
- Context: clinical monitoring, lifestyle interventions, pharmacological treatments, and multidisciplinary care strategies relevant to psychiatric and metabolic health.

A targeted literature search was conducted using the PubMed database, covering publications from January 2000 to June 2025. PubMed was selected due to its comprehensive coverage of biomedical and psychiatric literature and its indexing of major clinical and translational research in psychiatry and metabolic medicine.

The search strategy combined keywords and Medical Subject Headings (MeSH) across several thematic domains:

- Psychiatric conditions: “bipolar disorder”, “mood disorder”.
- Metabolic health: “metabolic syndrome”, “obesity”, “insulin resistance”, “cardiometabolic risk”.
- Biological mechanisms: “inflammation”, “cytokines”, “HPA axis”, “genetic polymorphisms”.
- Treatment-related factors: “antipsychotics”, “weight gain”, “GLP-1 receptor agonists”, “ketogenic diet”, “bariatric surgery”.
- Lifestyle interventions: “diet”, “physical activity”, “therapeutic patient education”

Eligible studies included peer-reviewed human studies, such as observational studies, clinical trials, systematic reviews, and meta-analyses addressing the association between BD and metabolic health or interventions targeting metabolic risk in this population.

Studies were excluded if they:

- involved only animal models,
- did not specifically address bipolar disorder or metabolic outcomes,
- or consisted solely of conference abstracts without full data.

Titles and abstracts were screened for relevance, followed by full-text review when necessary to ensure alignment with the PCC framework.

Although this review was not conducted as a systematic review, we sought to enhance transparency and methodological rigor by adhering to the SANRA (Scale for the Assessment of Narrative Review Articles) guidelines (22), which assess narrative reviews across six domains including justification of the review’s importance, literature search transparency, referencing quality, and scientific reasoning.

A summary of the literature search approach is presented in **Table 1**.

**Table 1:** Literature identification strategy used in the narrative review.

Step	Description
Database searched	PubMed
Time period	2000–June 2025
Population	People living with bipolar disorder
Concept	Metabolic syndrome and cardiometabolic risk
Context	Monitoring, lifestyle interventions, pharmacological and surgical management
Study types included	Clinical trials, observational studies, systematic reviews, meta-analyses
Exclusion criteria	Animal studies, non-relevant metabolic outcomes, conference abstracts

## 3 Results

### 3.1 Bidirectional relationship underlying the comorbidity

#### 3.1.1 Shared behavioral and clinical pathways

The association between BD and MetS is increasingly recognized as bidirectional. Several behavioral and clinical mechanisms may contribute to this relationship. On one hand, individuals with BD may have a higher likelihood of developing metabolic disturbancesdue to a combination of lifestyle-related factors, such as physical inactivity, emotional eating, smoking, and disrupted sleep patterns, exacerbated during mood episodes (23–25). In addition, long-term use of certain psychotropic medications, especially second-generation antipsychotics and mood stabilizers, is associated withweight gain, insulin resistance, and lipid abnormalities (18, 26–28).

On the other hand, the presence of MetS may negatively influence the clinical course of BD. Metabolic abnormalities, including insulin resistance and systemic inflammation, have been associated with increased severity and frequency of mood episodes, cognitive dysfunction, and reduced treatment response (29–33). Evidence suggests that MetS may interfere with neuroplasticity and neurotransmitter systems, which could contribute to worsening psychiatric symptoms (30, 34). Together, these mechanisms suggest the existence of a reinforcing relationship between metabolic and psychiatric processes, highlighting the importance of early detection and integrated management strategies.

#### 3.1.2 Inflammatory mechanisms

Chronic low-grade inflammation is increasingly recognized as a biological link between BD and MetS (32, 33, 35, 36). PlwBD exhibit elevated levels of pro-inflammatory cytokines, including interleukin-1 (IL-1), interleukin 6 (IL-6), tumor necrosis factor-alpha (TNF-α), and high-sensitivity C-reactive protein (hs-CRP), even during euthymic states (32, 36). These inflammatory markers have also been implicated in insulin resistance, a key driver of MetS (33).

These cytokines can cross the blood-brain barrier and activate microglial cells, leading to neuroinflammation. This has been linked to altered glutamate neurotransmission and excitotoxicity (37–39) as well as dysregulation of monoamines (dopamine, serotonin, norepinephrine) (35, 40, 41) and reduced neurotrophic support, particularly decreased BDNF, a key molecule for synaptic plasticity and neuronal survival (41–43). Low BDNF is a robust biomarker in BD and is particularly low during depressive and manic phases.

Together, these inflammatory processes may contribute both to mood dysregulation and to metabolic disturbances, reinforcing the biological links between BD and MetS.

#### 3.1.3 HPA axis dysregulation and cortisol

Another important biological pathway linking BD and MetS involves dysregulation of the hypothalamic–pituitary–adrenal (HPA) axis. Elevated cortisol levels are frequently observed in individuals with BD and have been associated with mood instability, cognitive impairment, and reductions in hippocampal volume (44, 45). These neurotoxic effects may contribute to both affective symptomatology and impaired emotion regulation (46). In parallel, sustained hyperactivation of the HPA axis plays a critical role in the development of MetS. Chronic cortisol elevation promotes central adiposity, increases hepatic gluconeogenesis, and induces insulin resistance, all of which are key components of the MetS (47). Additionally, cortisol influences lipid metabolism and blood pressure regulation, further aggravating dyslipidemia and hypertension (47).

Taken together, HPA axis dysregulation represents a shared neuroendocrine pathway linking chronic stress, metabolic dysfunction, and mood disorder progression.

#### 3.1.4 Metabolic effects of psychotropic medications

Pharmacological treatment remains essential in the management of BD, yet many psychotropic agents, particularly second-generation antipsychotics and certain mood stabilizers, are associated with significant metabolic side effects. These include weight gain, insulin resistance, dyslipidemia, and increased risk of type 2 diabetes, all of which contribute to the development or worsening of MetS (27, 48–50).

Second-generation antipsychotics such as olanzapine and clozapine have been consistently associated with the greatest metabolic burden (51–53). These medications may increase appetite by antagonizing histamine H1 and serotonin 5-HT2C receptors, interfere with glucose metabolism, and promote central fat accumulation through dysregulation of hypothalamic signaling and activation of the endocannabinoid system (54). Additionally, these agents may impair insulin sensitivity independently of weight gain.

Mood stabilizers also impact metabolic function. Valproate, for instance, has been associated with increased adiposity and altered lipid profiles (55). Although lithium treatment has been associated with altered thyroid function, current evidence suggests that these changes are often subclinical and not directly responsible for metabolic disturbances or significant weight gain. In fact, lithium may be metabolically neutral or even protective compared to other mood stabilizers (56). Even some antidepressants—especially tricyclics—have been linked to weight gain and insulin resistance (57–59).

Given the chronic nature of BD and the long-term use of these medications, clinicians must carefully balance psychiatric efficacy with metabolic safety. Systematic metabolic monitoring and careful selection of medications with lower cardiometabolic risk profiles are therefore essential components of comprehensive care.

#### 3.1.5 Genetic variation and antipsychotic-induced weight gain

While early research suggested that polymorphisms in the FTO gene commonly linked to obesity in the general population might contribute to metabolic side effects in PlwBD treated with antipsychotics (60), more recent studies have not consistently supported this association. In particular, investigations into the rs9939609 variant have shown no significant correlation with body mass index (BMI), waist circumference, or lipid abnormalities in people with schizophrenia or BD undergoing psychotropic treatment (61–63). These findings suggest that the contribution of FTO polymorphisms to antipsychotic-related metabolic disturbances may be limited. Instead, emerging research points toward the involvement of other pathways, including genes regulating insulin signaling, inflammatory responses, and mitochondrial function, which may offer more promising insights into the shared vulnerability between BD and metabolic disturbances (64–68).

Although genetic susceptibility may influence individual responses to psychotropic medications, current evidence remains insufficient to support routine genetic screening in clinical practice. Further research is needed to clarify the complex genetic architecture underlying metabolic risk in people living with BD.

**Figure 1:**
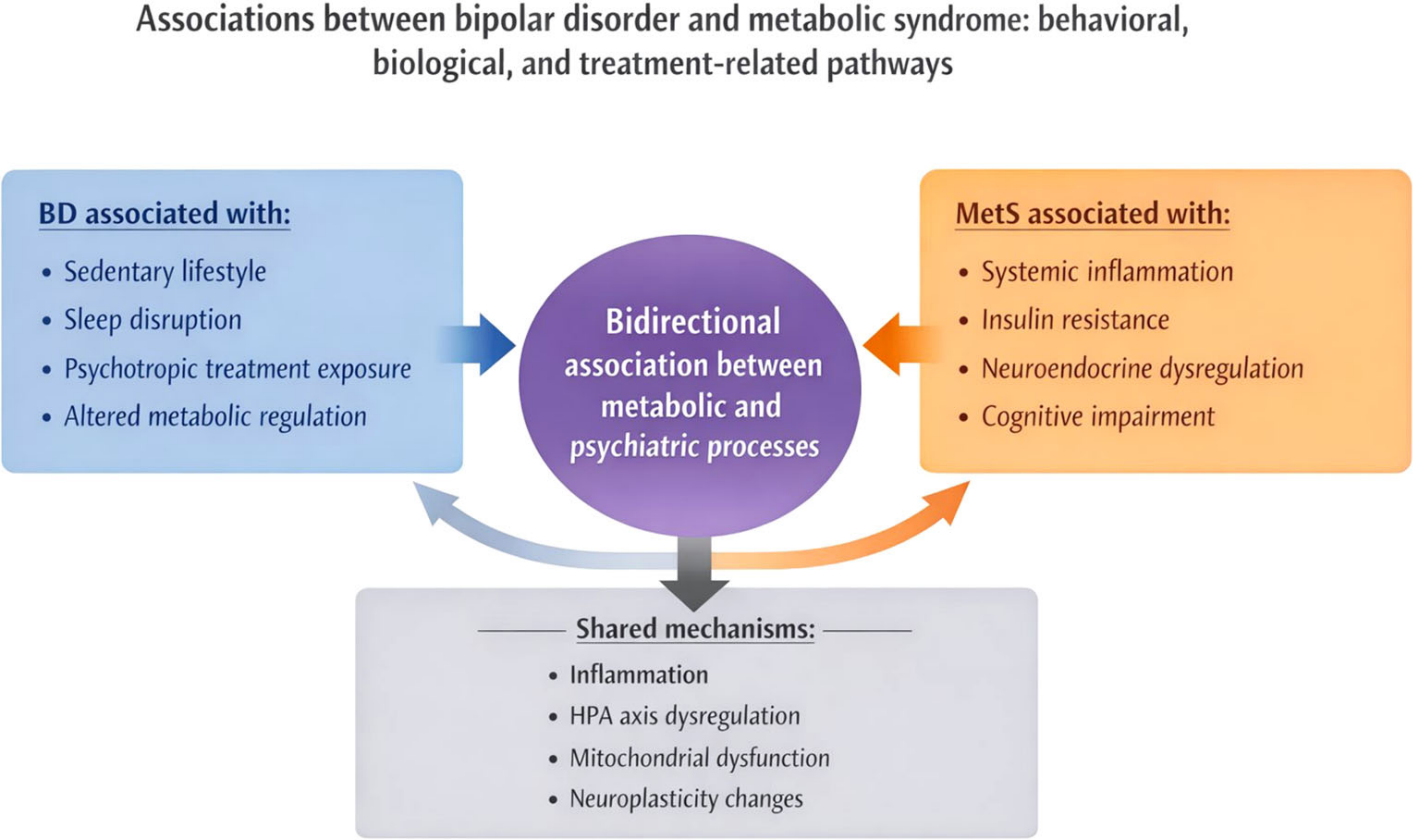
Bidirectional associations between bipolar disorder and metabolic syndrome.

A summary of the bidirectional link between BD and MetS is shown in **Figure 1**.

Bipolar disorder and metabolic syndrome frequently co-occur and share multiple behavioral, biological, and treatment-related pathways. Lifestyle factors, psychotropic medications, and metabolic abnormalities are associated with increased cardiometabolic risk in people living with bipolar disorder. Conversely, metabolic disturbances such as insulin resistance, systemic inflammation, and neuroendocrine dysregulation have been associated with mood instability and cognitive impairment. Together, these interconnected processes may contribute to the reciprocal relationship between psychiatric and metabolic health.

### 3.2 Metabolic monitoring and management of metabolic syndrome in bipolar disorder

#### 3.2.1 Challenges and gaps in guideline integration for bipolar and metabolic comorbidity

Despite the well-established bidirectional relationship between serious mental illness and cardiometabolic disease, integration of care remains limited. A recent guideline review by Silverstein et al., 2024 found that management recommendations are far more frequently included in psychiatric guidelines than psychiatric guidance appears in cardiovascular guidelines (69). Notably, European psychiatric societies, such as NICE and BAP, provide more comprehensive recommendations for baseline and ongoing monitoring of cardiometabolic risk such as blood pressure, weight, glycemic control, lipid profiles, and ECG, than their American counterparts (69).

Conversely, cardiovascular guidelines—particularly those from American societies rarely include recommendations regarding psychiatric comorbidities (69). This lack of reciprocity reflects persisting gaps in awareness among cardiovascular professionals regarding the impact of psychiatric disorders, including BD, on cardiometabolic outcomes.

However, developing truly comprehensive guidelines for BD is challenging due to the disorder’s heterogeneity, the sheer volume of treatment modalities (pharmacological, psychosocial), and the predominance of fragmented, expert-opinion–based evidence complicate synthesis into unified algorithms (69). Applying this critique to metabolic–psychiatric comorbidity underscores the difficulty of formulating integrated guidance that simultaneously addresses both psychiatric stability and cardiometabolic risk. Strengthening collaboration between psychiatry, primary care, and metabolic specialists therefore represents a key priority for improving long-term outcomes in this population.

#### 3.2.2 Metabolic monitoring

Given the elevated risk of cardiometabolic complications in individuals with BD, systematic and regular metabolic monitoring is essential. Clinical guidelines recommend routine assessments of weight, body mass index (BMI), waist circumference, blood pressure, fasting glucose, and lipid profiles in all PlwBD receiving psychotropic medications, particularly those at higher metabolic risk (70).

Monitoring should ideally begin before initiation of pharmacological treatment and continue regularly thereafter. For most parameters, assessments should be conducted at baseline, 3 months, and then every 6 to 12 months, depending on individual risk and treatment type (70, 71). For example:

- Weight, BMI, and waist circumference should be measured at each psychiatric follow-up.
- Blood pressure should be assessed at least annually or more frequently in hypertensive individuals.
- Fasting plasma glucose and lipid panel should be monitored every 6 to 12 months.

Regular monitoring of metabolic parameters should be complemented by treatment-specific safety assessments that may also have metabolic relevance. Thyroid function should be checked periodically in people with bipolar disorder and comorbid metabolic syndrome, as thyroid dysfunction contributes to weight fluctuations, obesity, and worsening metabolic risk. This is particularly important in patients receiving lithium, which is known to affect thyroid physiology. In addition, renal function should be assessed regularly in lithium-treated patients, as chronic kidney impairment may further complicate metabolic management.

For patients receiving valproate, hepatic function and platelet counts should be monitored periodically to detect potential hepatotoxicity or thrombocytopenia. While platelet monitoring is required primarily for treatment safety, hepatic assessment is relevant both to detect valproate-induced hepatotoxicity and to screen for metabolic dysfunction–associated steatotic liver disease (MASLD), a frequent complication of obesity and metabolic syndrome. This highlights the importance of integrating metabolic screening into routine psychiatric follow-up.

#### 3.2.3 Nutritional Interventions in bipolar disorder with metabolic syndrome

Nutritional strategies are fundamental to the management of MetS in individuals with BD. A 2022 systematic review by Gabriel et al. synthesized findings from 60 studies and demonstrated that dietary quality influences both mood stability and cardiometabolic health (72). Specifically, higher intake of omega-3 fatty acids, particularly EPA and DHA, was associated with reduced depressive symptoms and improved inflammatory profiles (72). Similarly, micronutrients such as folate, zinc, and coenzyme Q10 were linked to improved mood and potential benefits for mitochondrial and metabolic function (72). Probiotics also showed promise, likely through modulation of the gut-brain axis and systemic inflammation (72).

Beyond individual nutrients, broader dietary patterns—notably those resembling the Mediterranean or DASH diets—were associated with better mood outcomes, lower rates of MetS, and reduced cardiovascular risk. These diets emphasize fruits, vegetables, whole grains, fish, and healthy fats, while limiting processed foods, added sugars, and saturated fats (72).

Importantly, the review underlined that whole-diet approaches may yield greater benefits than isolated supplementation due to synergistic effects on metabolic and neuropsychiatric pathways (72). These findings support the integration of structured, evidence-based dietary counseling into multidisciplinary treatment plans for PlwBD, particularly those at risk of or living with MetS.

In addition to conventional approaches, emerging evidence supports the potential utility of the ketogenic metabolic therapy (KMT): a very low-carbohydrate, high-fat dietary intervention that induces a state of nutritional ketosis (73–75).

KMT has been an evidence-based treatment for drug-resistant epilepsy for more than a century, with multiple randomized trials and meta-analyses demonstrating its efficacy in reducing seizure frequency in both pediatric and adult populations (76–78). Interest in its potential psychiatric applications has grown in recent years due to its effects on neuronal excitability, mitochondrial function, and neuroinflammation—mechanisms that are also implicated in the pathophysiology of bipolar disorder. Several pilot studies and clinical observations have suggested that KMT may be feasible and potentially beneficial in individuals with bipolar disorder. For example, a recent pilot study reported that adherence to a ketogenic metabolic intervention for six to eight weeks was associated with reductions in depressive and manic symptoms, improvements in insulin sensitivity, and favorable changes in cerebral energy metabolism measured using magnetic resonance spectroscopy (79). Participants demonstrated improved metabolic activity in the anterior cingulate cortex, suggesting a potential neurometabolic effect of ketosis on mood regulation.

Mechanistically, ketogenic metabolic therapy has been proposed to i) enhance mitochondrial biogenesis and energy efficiency, ii) reduce oxidative stress and systemic inflammation, iii) modulate glutamatergic neurotransmission and iv) increase BDNF expression and neuroplasticity.

While these findings are promising, current evidence remains limited, as most available studies are small, short-term, and frequently uncontrolled (79–82). Adherence to KMT can also be challenging, particularly during depressive episodes characterized by low motivation or disorganized eating behaviors. Furthermore, potential adverse effects—including gastrointestinal symptoms, micronutrient deficiencies, and changes in lipid profiles—require careful monitoring and medical supervision.

Importantly, ketogenic metabolic therapy should not be considered a stand-alone intervention in the management of bipolar disorder with metabolic syndrome. Its safe and effective implementation requires integration into a multidisciplinary treatment framework that includes psychiatric monitoring, nutritional supervision, and lifestyle interventions.

First, individualized nutritional assessment is necessary to determine whether KMT is appropriate for a given patient, taking into account metabolic status, comorbidities, and readiness for dietary change. Psychiatric stability should also be evaluated before initiating this intervention.

Second, continuous monitoring is essential. This includes periodic evaluation of ketone levels, lipid profiles, renal and hepatic function, and psychiatric symptoms. Dietary changes may alter medication metabolism, necessitating close collaboration between psychiatrists, dietitians, and primary care clinicians.

Finally, structured follow-up and behavioral support are critical for sustaining adherence. Behavioral coaching, motivational interviewing, digital health tools, and psychoeducation may all facilitate long-term adherence and reduce relapse risk. Family involvement may further support successful implementation.

#### 3.2.4 Physical activity promotion in bipolar disorder with metabolic syndrome

Regular physical activity is a key component in the management of MetS (83) and has additional benefits for mood stabilization, cognitive functioning, and overall well-being in individuals with BD (76). Despite these advantages, levels of physical activity remain significantly lower in this population compared to the general public, due to motivational deficits, fatigue, fear of triggering mood episodes, and lack of tailored programs (77).

To be effective, physical activity interventions must be structured, progressive, and individualized. Moderate-intensity aerobic exercises—such as brisk walking, cycling, or swimming—performed at least 150 minutes per week are recommended, in line with WHO guidelines. Resistance training may also be beneficial, particularly in addressing sarcopenic obesity and improving insulin sensitivity.

Behavioral strategies such as goal setting, activity tracking with wearable devices, and motivational interviewing can improve engagement and adherence. Group sessions, peer support, and integration into psychosocial rehabilitation programs further increase feasibility and impact. Importantly, exercise prescriptions should be developed in collaboration with physiotherapists or exercise specialists familiar with psychiatric conditions, to ensure safety and promote long-term sustainability (78).

By addressing both physical and mental health goals, exercise-based interventions contribute not only to metabolic risk reduction but also to improved mood regulation, sleep quality, and functional recovery.

#### 3.2.5 Emerging treatments: GLP-1 receptor agonists

Glucagon-like peptide-1 receptor agonists (GLP-1 RAs), originally developed for the treatment of type 2 diabetes, have shown promising results in addressing obesity and metabolic complications in psychiatric populations (79, 80). These agents promote glucose-dependent insulin secretion, delay gastric emptying, and enhance satiety, resulting in significant weight loss and improvements in glycemic control (80).

In the context of BD, small-scale studies and case series have demonstrated that GLP-1 RAs such as liraglutide and semaglutide may mitigate antipsychotic-induced weight gain and metabolic dysregulation, particularly in PlwBD resistant to lifestyle interventions or metformin therapy (81, 82, 84, 85). Notably, a randomized controlled trial in individuals with severe mental illness treated with clozapine or olanzapine reported that adjunctive liraglutide therapy led to significant reductions in body weight and HbA1c over 16 weeks, without destabilizing psychiatric symptoms (86).

Beyond metabolic benefits, preliminary evidence suggests potential neuroprotective and anti-inflammatory properties of GLP-1 RAs, which may confer additional advantages for mood regulation and cognitive function (80), though more research is needed to confirm these effects in bipolar populations.

GLP-1 RAs are generally well-tolerated, with gastrointestinal symptoms being the most common side effects. However, they should never be used as a stand-alone solution. For sustained benefit, GLP-1 RAs must be embedded in a comprehensive lifestyle-oriented care plan, including tailored nutritional guidance, physical activity support, and TPE. Their effectiveness depends not only on pharmacologic mechanisms but also on behavioral engagement and long-term adherence, which require multidisciplinary coordination and patient empowerment.

#### 3.2.6 Bariatric surgery: a consideration for severe obesity in bipolar disorder

Bariatric surgery is an effective intervention for individuals with severe obesity and comorbid MetS, leading to substantial and sustained weight loss, remission of type 2 diabetes, and reduction in cardiovascular risk (87). In the general population, it is associated with significant improvements in quality of life and long-term survival. Its role in PlwBD, however, has traditionally been met with caution due to concerns over psychiatric instability and adherence to postoperative care.

A large prospective study found that approximately two-thirds of bariatric surgery candidates met criteria for a lifetime DSM-IV Axis I psychiatric disorder, and nearly 40% met criteria for a current diagnosis at the time of preoperative assessment (88). Mood disorders, including BD, were among the most common conditions identified (88). These findings highlight that psychiatric comorbidity is not uncommon among surgical candidates, and that BD in itself should not be viewed as an exclusion criterion. Importantly, authors also observed that when individuals with psychiatric comorbidities received comprehensive psychological support, they achieved postoperative outcomes—including weight loss and functional improvement—comparable to those without psychiatric diagnoses (88). Rates of psychiatric decompensation following surgery were also relatively low, reinforcing the feasibility of bariatric interventions in this population when delivered within a structured, supportive care model.

Friedman et al. further clarified this picture: of bipolar-spectrum PlwBD seeking bariatric surgery, 57% received psychological approval, and 48% underwent the procedure (89). Those selected for surgery generally had fewer prior psychiatric hospitalizations. Although follow-up attendance declined beyond two years, weight loss outcomes at 12-month and longer-term follow-up were comparable to matched controls without BD, demonstrating durable metabolic benefit without increased risk of psychiatric deterioration (89). These results are in alignment with more recent studies (90).

Eligibility for surgery should follow standard criteria (e.g., BMI ≥35 kg/m2 or ≥30 kg/m2 with comorbidities) (91), but additional emphasis must be placed on mental health stability, medication adherence, and availability of long-term psychiatric and nutritional support. Preoperative evaluations should include a comprehensive psychiatric assessment to ensure mood stability and readiness for the behavioral demands of the procedure. Postoperative care must be integrated into a multidisciplinary framework to address both physical and psychological needs, including monitoring for potential mood fluctuations and ensuring nutritional adequacy.

#### 3.2.7 Therapeutic patient education and patient-centered care

Therapeutic Patient Education (TPE) plays a pivotal role in empowering individuals BD and MetS to manage both mental and physical health actively. TPE extends beyond imparting information—it encompasses enhancing self-management abilities, fostering autonomy, and strengthening coping strategies within a comprehensive, biopsychosocial framework (92).

A recent systematic review of TPE in severe mental illness was conducted, identifying 44 programs across diagnoses including BD (93). While outcomes varied, several trials reported significant improvements in symptom control, self-efficacy, and everyday functioning (93). Notably, psychoeducational programs tailored to BD have consistently demonstrated enhanced treatment adherence, reduced relapse rates, and improved quality of life (93). For example, Lequimener-de Lorgeril et al. (2019) observed increased self-esteem and trends toward better quality-of-life scores following an eight-session bipolar-specific TPE program (94). These findings are supported by multiple randomized controlled trials indicating that psychoeducation decreases hospitalization and increases time to relapse in PlwBD (95).

Central to TPE’s success is a patient-centered, collaborative approach that respects individual preferences, tailors content to readiness and lived experience, and integrates peer support (92). This approach promotes empowerment, hope, and connectedness among participants, core elements of recovery. Peer involvement enriches the learning environment, reducing stigma and modeling effective coping strategies. Given the chronic, fluctuating course of both BD and MetS, TPE delivered through a sustained, multidisciplinary framework, incorporating psychiatry, nutrition, behavioral therapy, and primary care, enables patients to become active, informed partners in their health.

In essence, TPE fosters therapeutic alliance, improves engagement, and bridges the gap between clinical recommendations and real-world self-management. By nurturing patient agency and reinforcing skills across behavioral, medical, and psychological domains, TPE directly contributes to improved treatment adherence, mood stability, and long-term metabolic health.

**Figure 2:**
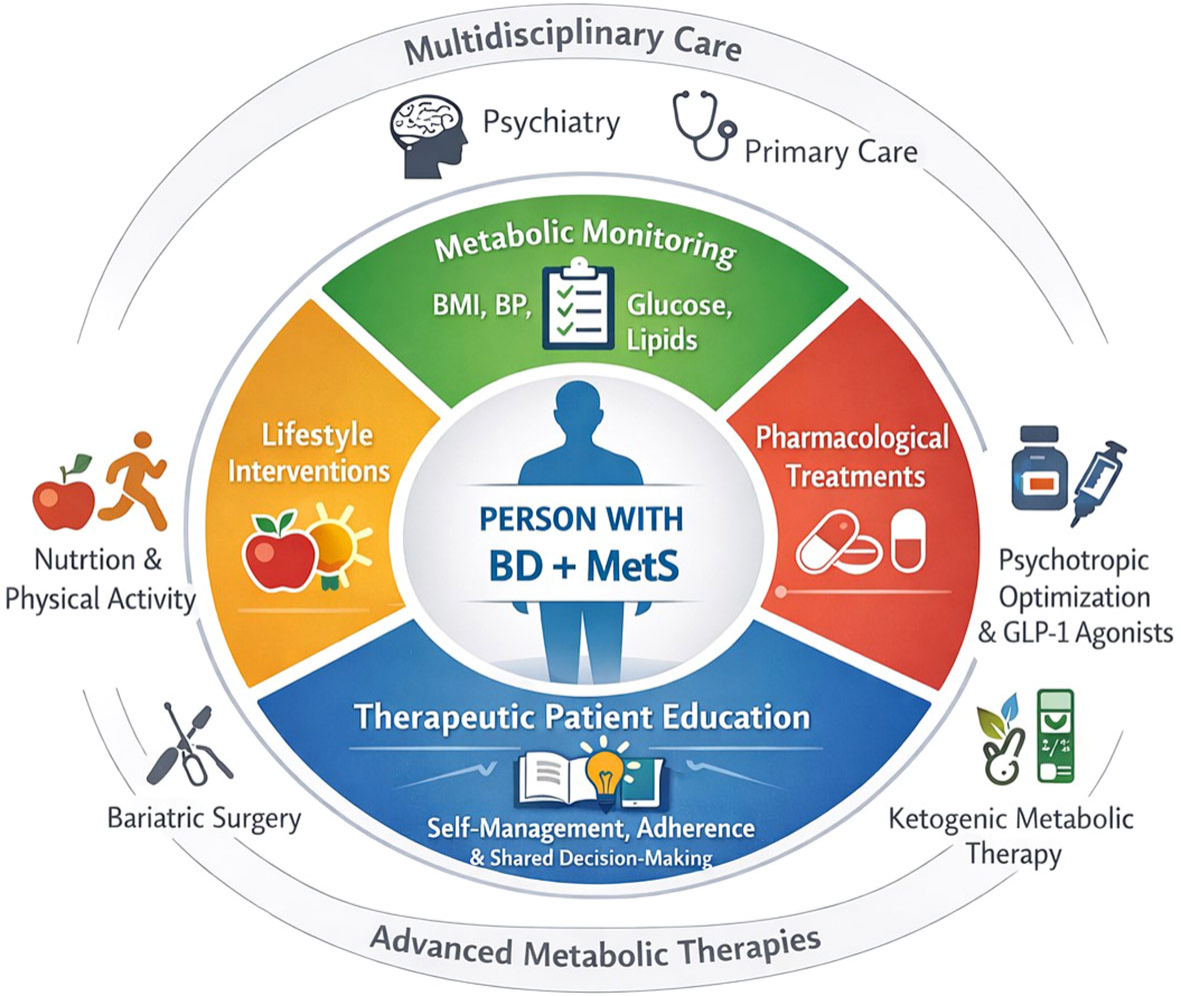
Multidisciplinary management of metabolic syndrome in people living with bipolar disorder.

The different axes for managing MetS in PlwBD are summarized in **Figure 2**.

Management of metabolic syndrome in bipolar disorder requires a patient-centered and multidisciplinary approach. Core domains include systematic metabolic monitoring, lifestyle interventions (nutrition and physical activity), pharmacological strategies such as GLP-1 receptor agonists, and therapeutic patient education to support self-management and treatment adherence. In selected patients with severe or refractory obesity, advanced metabolic therapies such as bariatric surgery or ketogenic metabolic therapy may be considered within specialized care pathways.

## 4 Limitations and implications

This narrative review has several limitations. First, the literature search was restricted to a single database (PubMed). Although this database provides broad coverage of biomedical and psychiatric research, the exclusion of other sources (e.g., Embase, PsycINFO, Cochrane) may have limited the comprehensiveness of the review and introduced selection bias.

Second, as a narrative review, this work does not follow a fully systematic methodology. Despite efforts to enhance rigor and transparency, including the use of the SANRA framework, the selection and synthesis of studies may be subject to author interpretation and do not provide quantitative estimates of effect.

Third, the evidence supporting several emerging interventions—such as ketogenic metabolic therapy, GLP-1 receptor agonists in psychiatric populations, and bariatric surgery in individuals with bipolar disorder remains limited. Available studies are often characterized by small sample sizes, short follow-up periods, and methodological heterogeneity, limiting the strength and generalizability of conclusions.

These limitations have important clinical implications. Lifestyle interventions remain the cornerstone of cardiometabolic risk reduction and should be systematically implemented. Pharmacological and surgical strategies should be considered as adjunctive options within a structured, multidisciplinary care framework.

Future research should prioritize well-designed longitudinal and interventional studies in bipolar populations, as well as the development of integrated clinical guidelines bridging psychiatric and metabolic care.

## 5 Conclusion

The coexistence of BD and MetS represents a complex and clinically significant challenge, driven by shared biological pathways, adverse effects of psychotropic medications, and behavioral vulnerabilities. This comorbidity not only heightens the risk of cardiovascular morbidity and mortality but also compromises psychiatric stability, cognitive function, and overall quality of life.

Effective management requires a proactive, multidisciplinary approach that integrates psychiatric care with metabolic monitoring, lifestyle interventions, and TPE. Emerging strategies, including GLP-1 receptor agonists and bariatric surgery in selected patients, may further enhance treatment options when embedded within this broader care model. Ultimately, improving outcomes in this population will depend on strengthening collaboration across disciplines and embedding cardiometabolic prevention and management into routine psychiatric care. Such an approach holds the potential to reduce health disparities and support sustained recovery in people living with bipolar disorder.
